# The effects of object size on spatial orientation: an eye movement study

**DOI:** 10.3389/fnins.2023.1197618

**Published:** 2023-11-10

**Authors:** Tianqi Yang, Yang He, Lin Wu, Hui Wang, Xiuchao Wang, Yahong Li, Yaning Guo, Shengjun Wu, Xufeng Liu

**Affiliations:** ^1^Department of Military Medical Psychology, Air Force Medical University, Xi’an, China; ^2^Central Theater Command Air Force Hospital of PLA, Datong, China

**Keywords:** spatial orientation, object size, eye movement, cognitive load, cognitive penetrability

## Abstract

**Introduction:**

The processing of visual information in the human brain is divided into two streams, namely, the dorsal and ventral streams, object identification is related to the ventral stream and motion processing is related to the dorsal stream. Object identification is interconnected with motion processing, object size was found to affect the information processing of motion characteristics in uniform linear motion. However, whether the object size affects the spatial orientation is still unknown.

**Methods:**

Thirty-eight college students were recruited to participate in an experiment based on the spatial visualization dynamic test. Eyelink 1,000 Plus was used to collect eye movement data. The final direction difference (the difference between the final moving direction of the target and the final direction of the moving target pointing to the destination point), rotation angle (the rotation angle of the knob from the start of the target movement to the moment of key pressing) and eye movement indices under conditions of different object sizes and motion velocities were compared.

**Results:**

The final direction difference and rotation angle under the condition of a 2.29°-diameter moving target and a 0.76°-diameter destination point were significantly smaller than those under the other conditions (a 0.76°-diameter moving target and a 0.76°-diameter destination point; a 0.76°-diameter moving target and a 2.29°-diameter destination point). The average pupil size under the condition of a 2.29°-diameter moving target and a 0.76°-diameter destination point was significantly larger than the average pupil size under other conditions (a 0.76°-diameter moving target and a 0.76°-diameter destination point; a 0.76°-diameter moving target and a 2.29°-diameter destination point).

**Discussion:**

A relatively large moving target can resist the landmark attraction effect in spatial orientation, and the influence of object size on spatial orientation may originate from differences in cognitive resource consumption. The present study enriches the interaction theory of the processing of object characteristics and motion characteristics and provides new ideas for the application of eye movement technology in the examination of spatial orientation ability.

## Introduction

Spatial orientation, defined as a natural ability to maintain body posture and orientation in relation to the environment when an individual is at rest or in motion (Meeks et al., 2022), is a core factor of the flight competency model. Appropriate spatial orientation depends on accurate perception and cognitive integration of the visual, vestibular, and proprioceptive systems (Medendorp et al., 2018). If the systems provide contradictory information, a sensory conflict will be experienced and will result in spatial disorientation (Meeks et al., 2022). Spatial disorientation can impair the cognitive function and the psychomotor performance of pilots, affecting their flight control (Gresty et al., 2003, 2008; Webb et al., 2012; Kowalczuk et al., 2016) and thereby seriously threatening flight safety (Newman and Rupert, 2020). According to previous reports, spatial disorientation contributes to more than 30% of aviation mishaps that have fatality rates of nearly 100% (Gibb et al., 2011; Hao et al., 2022).

In humans, approximately 80% of the sensory input that contributes to maintaining spatial orientation comes from visual information provided by the eyes (Meeks et al., 2022). The processing of visual information in the human brain is divided into two streams, namely, the dorsal and ventral streams (Saber et al., 2015; Takemura et al., 2016; Milner, 2017; Sani et al., 2019). From the primary visual cortices in the occipital lobe, visual information follows either the ventral or the dorsal stream (Chang et al., 2015; Micheletti et al., 2021). The ventral stream (the “what” pathway), which traverses the inferotemporal cortex, supports the identification of objects, while the dorsal stream (the “where” pathway) runs to the posterior parietal cortex and processes spatial information in the reference frame to coordinate movement (van Polanen and Davare, 2015). The functional segregation of object identification related to the ventral stream from motion processing related to the dorsal stream has been confirmed in a handful of studies (James et al., 2001, 2003; Milner and Goodale, 2006, 2008; Goodale and Milner, 2013). However, these two streams are not wholly independent (Saber et al., 2015; Milner, 2017; Janssen et al., 2018). In previous studies, researchers injected tracer fluids into the brains of monkeys and found connections between the parietal areas of the dorsal stream and the inferotemporal areas of the ventral stream (Borra et al., 2010). When transcranial magnetic stimulation was applied to parietal areas of the human brain, the ipsilateral middle temporal and fusiform gyri were found to be activated (Zanon et al., 2010). In addition, white matter tracts (the posterior vertical pathway) have been reported to directly bridge the cortical regions connected with the ventral and dorsal streams (Wu et al., 2016; Weiner et al., 2017; Bullock et al., 2019). Yan and You (2015) used a relative arrival time task to study differences between pilots and ordinary participants in the processing of the motion characteristics under different object characteristics. In ordinary participants, the size of the moving object was found to affect motion time estimation, reflecting the interaction of the processing of object characteristics with the processing of linear motion characteristics, whereas this interaction was greatly reduced in pilots. However, there was no orientation perception in the time estimation of linear motion, and as a passive perception process, it could not reflect the motion manipulation of individuals. Do the sizes of objects in motion affect spatial orientation? The present study is designed to address this question.

As suggested by Horner et al. (2019), although the accuracy of motion estimation is a common dependent variable in studies, it does not provide a good index from which to derive a complete understanding of the cognitive process. Eye movement tracking is a technique for statistically analyzing and visualizing the movements of the eyes (Kok and Jarodzka, 2017). Eye movement can reflect underlying cognitive processing in the brain and thus can be used in evaluating the perception and cognitive abilities of individuals (Hsiao et al., 2021); in addition, it is a fast response to external stimuli and is difficult to disguise (Yin et al., 2022). The advantages of eye movement technology make it widely used in studies of cognitive abilities (Makin and Poliakoff, 2011; de Sperati and Thornton, 2019; Chen et al., 2022). In view of this, eye movement technology was used in the present study to reveal how object characteristics affect spatial orientation in motion.

In summary, the present study compared the accuracy of spatial orientation and eye movement under conditions of different object sizes. This study could be of great significance in enriching the interaction theory of the ventral and dorsal stream, and it provides suggestions for the application of eye movement technology in selection of occupation and in training for spatial orientation ability.

## Participants and methods

### Participants

A total of 38 college students (age 22.32 ± 1.89 years) from Xi’an, China, were recruited to participate in the experiment. The recruitment criteria were male sex, right-handedness, normal vision or corrected-to-normal vision, no amblyopia or astigmatism, no history of sensory, perceptual or motor disorders, and no experience participating in similar experiments. All participants provided their consent before the experiment. The study was performed in accordance with the principles set forth in the 1991 Declaration of Helsinki and approved by the Ethics Committee of the Xijing Hospital of the Air Force Medical University (no. KY20224106-1).

### Methods

#### Apparatus

**Figure 1:**
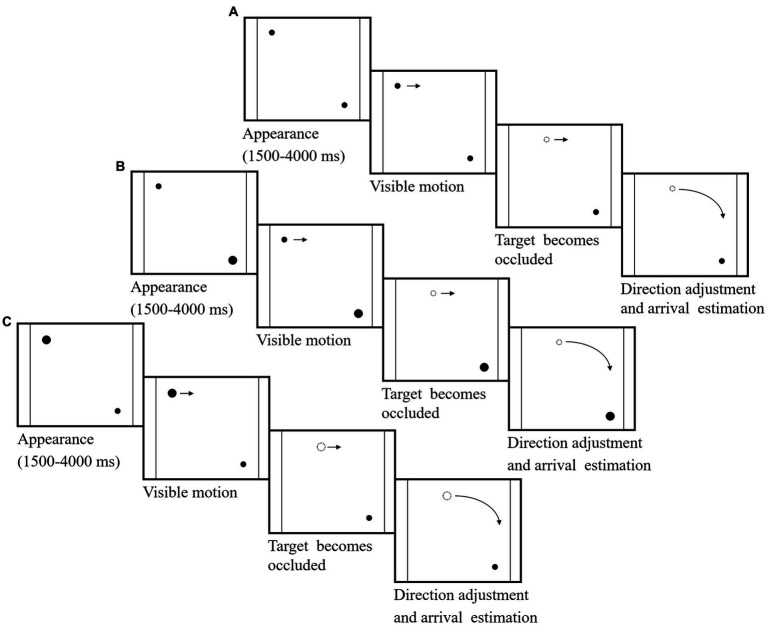
The experimental process. **(A)** The diameter of the moving target was 0.76°, and the diameter of the destination point was 0.76°. **(B)** The diameter of the moving target was 0.76°, and the diameter of the destination point was 2.29°. **(C)** The diameter of the moving target was 2.29°, and the diameter of the destination point was 0.76°.

The visual stimuli were presented on a Dell p1917s display (length 37.5 cm and width 30 cm) with a refresh rate of 60 Hz. The eye movement data were collected by Eyelink 1,000 Plus, the sampling rate was set to 1,000 Hz, and the headrest was fixed 75 cm from the screen. Experiment Builder software, version 2.2.61, was used to develop experimental programs based on the spatial visualization dynamic test (SVDT). As shown in Figure 1, three combinations of the diameters of the moving target and the destination point were used (moving target 0.76° and destination point 0.76°; moving target 0.76° and destination point 2.29°; moving target 2.29° and destination point 0.76°). The moving target and the destination point were, respectively, located in the upper left corner and the lower right corner of a square area (edge length 30 cm) at a distance of 25.50° from each other. After a random time between 1,500 ms and 4,000 ms from the start of the session, the moving target began to move horizontally to the right at a constant velocity (slow, 7.63°/s or fast, 11.42°/s). After the target had moved 3.82°, it was occluded but kept moving, and its velocity remained unchanged. The participants were required to adjust the direction of movement of the target clockwise using a circular knob with a diameter of 4 cm and to press the space bar when they estimated that the moving target had reached the destination point. The center of the moving target was not permitted to touch the edges of the square area, and the time for each trial could not exceed 10 s.

#### Experimental design

A within-subjects design with two factors was adopted in the present study. The independent variables were the diameters of the moving target and the destination point (moving target 0.76° diameter and destination point 0.76° diameter; moving target 0.76° diameter and destination point 2.29° diameter; moving target 2.29° diameter and destination point 0.76° diameter) and the velocity of the moving target (slow, 7.63°/s; fast, 11.42°/s). The two velocities were used randomly in the individual trials to prevent the participants from preparing for the velocity of motion in advance. The dependent variables were as follows: (1) the difference between the moving direction of the target and the direction of the moving target pointing to the destination point when the participants estimated that the moving target had reached the destination point and pressed the space key (referred to as the direction difference), reflecting the accuracy of the participant’s adjustment of the moving target’s direction. When participants excessively adjust the knob clockwise, the moving direction of the target is in the clockwise direction of the direction of the moving target pointing towards the destination point, the direction difference is positive. When participants insufficiently adjust the knob clockwise, the moving direction of the target is in the counterclockwise direction of the direction of the moving target pointing towards the destination point, the direction difference is negative. (2) the rotation angle of the knob from the start of the target movement to the moment of key pressing when the participants estimated that the moving target had reached the destination point (referred to as rotation angle). (3) the average pupil size of the participant from the start of the target movement to the moment of key pressing when the participants estimated that the moving target had reached the destination point (referred to as average pupil size). (4) the fixation count from the start of the target movement to the moment of key pressing when the participants estimated that the moving target had reached the destination point (referred to as fixation count). (5) the saccade count from the start of the target movement to the moment of key pressing when the participants estimated that the moving target had reached the destination point (referred to as saccade count). (6) the average duration of fixation from the start of the target movement to key pressing when the participants estimated that the moving target had reached the destination point (referred to as average fixation duration). (7) the average saccade amplitude from the start of the target movement to key pressing when the participants estimated that the moving target had reached the destination point (referred to as average saccade amplitude).

#### Procedure

The first stage was the practice test, and throughout the entire process of the practice test, the moving target was visible, thus participants were able to clearly understand the corresponding relationship between knob rotation and changes in the direction of the moving target. The Eyelink camera was focused to obtain the sharpest possible image of the participant’s eyes. The thresholds of pupil and corneal reflection (CR) were set to effectively distinguish the eyes from other areas of the image. Mapping the centers of the pupil and the CR helped in estimating the gaze direction (Nyström et al., 2023). Systematic 9-point calibration and validation were then performed to determine the correspondence between the position of the pupil-CR in the camera image and the gaze position on the display screen. Subsequently, the test began: each trial with the combination of different velocities and different diameters of the moving target and the destination point was presented 5 times, and a total of 30 trials were presented randomly.

The second stage was the formal test. Each trial with the combination of different velocities and different diameters of the moving target and the destination point was presented 10 times, and a total of 60 trials are presented randomly. Other procedures were the same as those used in the practice test.

### Statistical analysis

Data Viewer software, version 4.2.1, was used to package and export the information on the timing and position of the eye movements and keys. SPSS software, version 22.0, was used in the statistical analysis. Two-way repeated measures analysis of variance was used to test the effects of the diameters of the moving target and the destination point and the velocity of the moving target on direction difference, rotation angle and the eye movement indices. *Post hoc* analysis was conducted following the rejection of an omnibus null hypothesis. For the above statistical analyses, the significance level was taken as α = 0.05.

## Results

### Demographic characteristics of the participants

The age of the participants was 22.32 ± 1.89 years. The proportion of only children among the participants was 31.58%, and 57.89% of the participants were from urban areas. Undergraduate students accounted for 84.21% of the participants, and postgraduate students accounted for 15.79%.

### Effects of object diameter and velocity on direction difference

**Figure 2:**
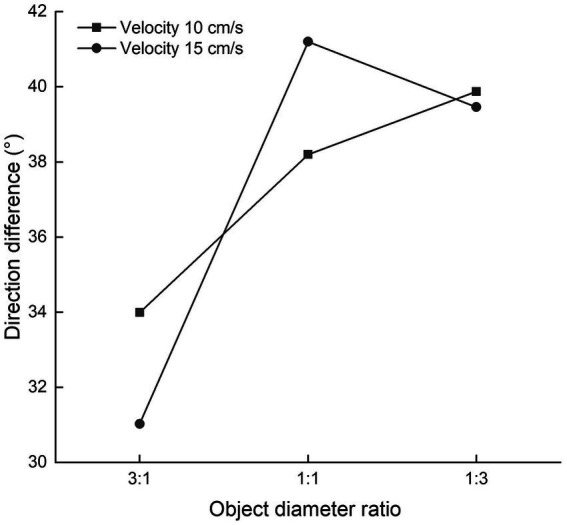
Effects of object diameter and velocity on direction difference.

The effects of object diameter and velocity on direction difference are displayed in Figure 2. The effect of object diameter on direction difference was significant, and the distribution failed to pass Mauchly’s test of sphericity, so the Greenhouse–Geisser method was used (*F* = 3.547, *p* = 0.034, *η*^2^ = 0.087). *Post hoc* analysis revealed that the direction difference when the moving target was 2.29° in diameter and the destination point was 0.76° in diameter was significantly smaller than the direction difference under other conditions (*p* = 0.004 for moving target diameter 0.76° and destination point diameter 0.76°; *p* = 0.045 for moving target diameter 0.76° and destination point diameter 2.29°). The effect of the velocity of the moving target on direction difference was not significant (*F* = 0.002, *p* = 0.967, *η*^2^ < 0.001). The interaction of object diameter with the velocity of the moving target was not significant (*F* = 0.678, *p* = 0.511, *η*^2^ = 0.018).

### Effects of object diameter and velocity on rotation angle

**Figure 3:**
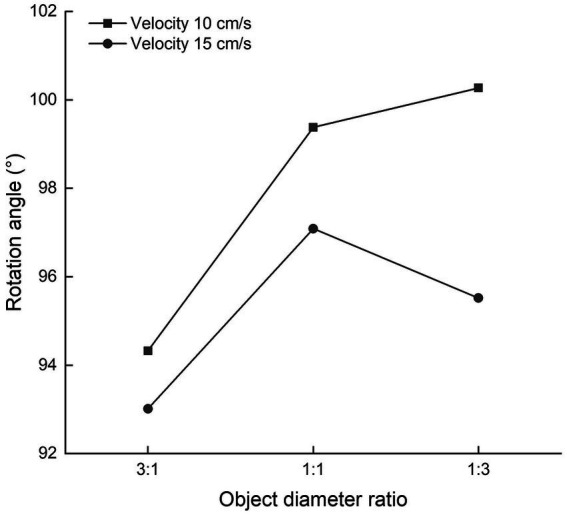
Effects of object diameter and velocity on rotation angle.

The effects of object diameter and velocity on the rotation angle are displayed in Figure 3. The effect of object diameter on rotation angle was significant, and the distribution failed to pass Mauchly’s test of sphericity, so the Greenhouse–Geisser method was used (*F* = 5.577, *p* = 0.009, *η*^2^ = 0.131). *Post hoc* analysis revealed that the rotation angle when the moving target was 2.29° in diameter and the destination point was 0.76° in diameter was significantly smaller than the rotation angle under other conditions (*p* = 0.013 for moving target diameter 0.76° and destination point diameter 0.76°; *p* = 0.011 for moving target diameter 0.76° and destination point diameter 2.29°). The effect of the velocity of the moving target on rotation angle was not significant (*F* = 1.820, *p* = 0.185, *η*^2^ = 0.047). The interaction of object diameter with the velocity of the moving target was not significant (*F* = 0.897, *p* = 0.412, *η*^2^ = 0.024).

### Effects of object diameter and velocity on eye movement indices

**Figure 4:**
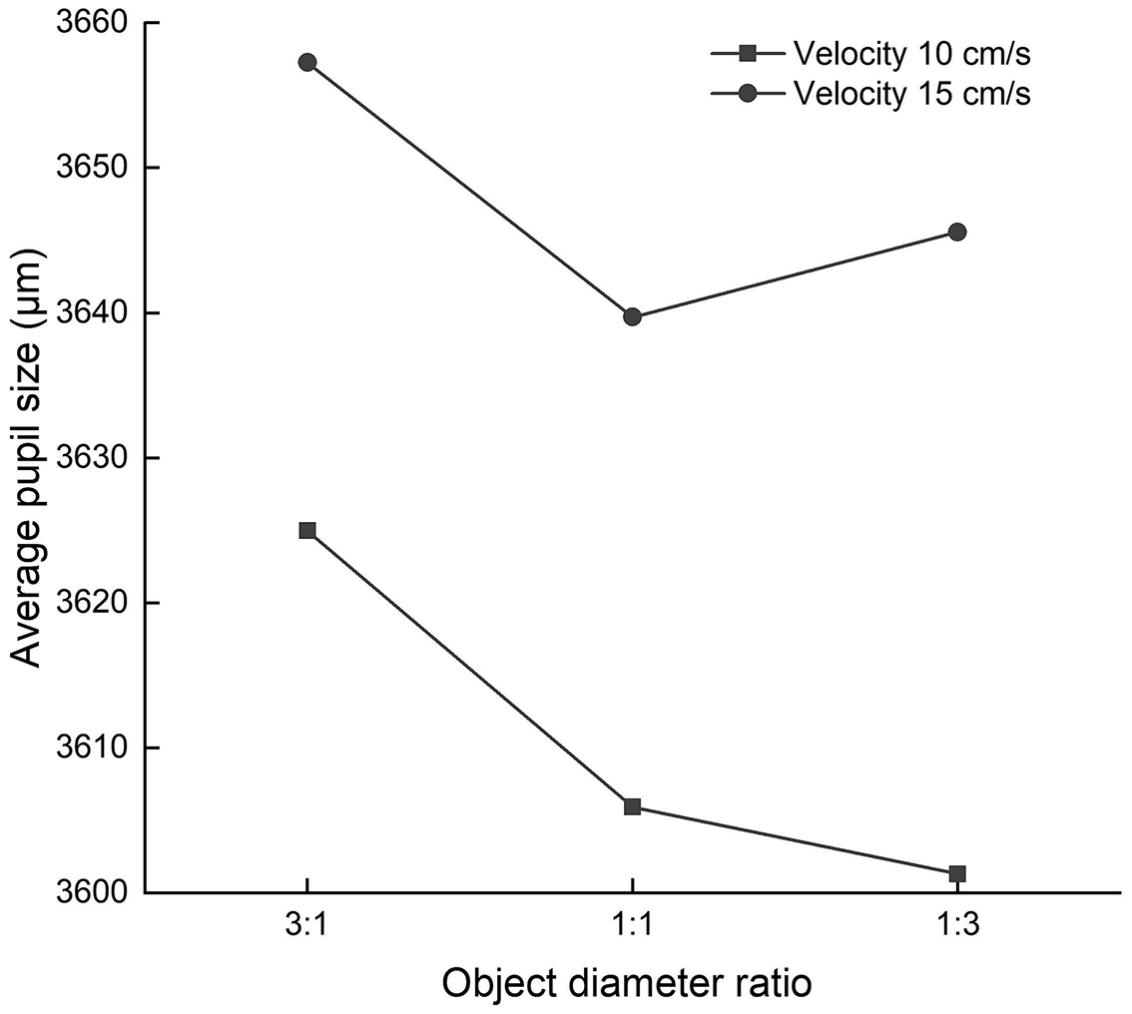
Effects of object diameter and velocity on average pupil size.

The effects of object diameter and velocity on average pupil size are displayed in Figure 4. The effect of object diameter on average pupil size was significant (*F* = 3.330, *p* = 0.041, *η*^2^ = 0.083), and *post hoc* analysis revealed that the average pupil size when the moving target was 2.29° in diameter and the destination point was 0.76° in diameter was significantly larger than the average pupil size under other conditions (*p* = 0.045 for moving target diameter 0.76° and destination point diameter 0.76°; *p* = 0.040 for moving target diameter 0.76° and destination point diameter 2.29°). The effect of the velocity of the moving target on average pupil size was significant (*F* = 26.090, *p* < 0.001, *η*^2^ = 0.414). The interaction of object diameter with the velocity of the moving target was not significant (*F* = 0.681, *p* = 0.509, *η*^2^ = 0.018).

The means of fixation count, saccade count, average fixation duration and average saccade amplitude under different conditions of velocity and object diameter ratio can be found in Table 1.

**Table 1:** The means of fixation count, saccade count, average fixation duration and average saccade amplitude under different conditions of velocity and object diameter ratio.

Velocity	Object diameter ratio	Fixation count	Saccade count	Average fixation Duration (ms)	Average saccade Amplitude (°)
7.63°/s	3:1	6.88	6.00	378.39	4.27
	1:1	7.01	6.13	365.10	4.50
	1:3	6.74	5.86	370.78	4.45
11.42°/s	3:1	5.49	4.67	345.61	5.26
	1:1	5.39	4.54	332.25	5.75
	1:3	5.03	4.16	343.42	6.22

The effect of object diameter on fixation count was significant (*F* = 9.804, *p* < 0.001, *η*^2^ = 0.209), and *post hoc* analysis revealed that the fixation count when the moving target was 0.76° in diameter and the destination point was 2.29° in diameter was significantly smaller than that under other conditions (*p* = 0.002 for moving target diameter 2.29° and destination point diameter 0.76°; *p* < 0.001 for moving target diameter 0.76° and destination point diameter 0.76°). The effect of the velocity of the moving target on fixation count was significant (*F* = 139.991, *p* < 0.001, *η*^2^ = 0.791). The interaction of object diameter with the velocity of the moving target was not significant (*F* = 2.377, *p* = 0.100, *η*^2^ = 0.060).

The effect of object diameter on saccade count was significant (*F* = 10.489, *p* < 0.001, *η*^2^ = 0.221), and *post hoc* analysis revealed that the saccade count when the moving target was 0.76° in diameter and the destination point was 2.29° in diameter was significantly smaller than that under other conditions (*p* = 0.002 for moving target diameter 2.29° and destination point diameter 0.76°; *p* < 0.001 for moving target diameter 0.76° and destination point diameter 0.76°). The effect of the velocity of the moving target on saccade count was significant (*F* = 134.261, *p* < 0.001, *η*^2^ = 0.784). The interaction of object diameter with the velocity of the moving target was not significant (*F* = 2.966, *p* = 0.058, *η*^2^ = 0.074).

The effect of object diameter on the average fixation duration was not significant, and the distribution failed to pass Mauchly’s test of sphericity, so the Greenhouse–Geisser method was used (*F* = 2.437, *p* = 0.103, *η*^2^ = 0.062). The effect of the velocity of the moving target on average fixation duration was significant (*F* = 37.972, *p* < 0.001, *η*^2^ = 0.506). The interaction of object diameter with the velocity of the moving target was not significant (*F* = 0.141, *p* = 0.869, *η*^2^ = 0.004).

The effect of object diameter on average saccade amplitude was significant (*F* = 12.274, *p* < 0.001, *η*^2^ = 0.249). The effect of the velocity of the moving target on average saccade amplitude was also significant (*F* = 77.011, *p* < 0.001, *η*^2^ = 0.675). The interaction of object diameter with the velocity of the moving target was significant (*F* = 7.299, *p* = 0.001, *η*^2^ = 0.165).

## Discussion

In the current study, as the results indicate, individuals tended to underestimate the adjustment angle in spatial orientation when the moving target was relatively large. Hubbard and Ruppel (2000) found that when a target was presented for 1 s on either side of a larger landmark, the remembered target location of the participants showed a bias toward the landmark; this bias was called the “landmark attraction effect.” In addition, the characteristics of landmarks, such as size and situational meaning, also affect judgments of the position of moving targets (Hubbard and Ruppel, 1999; Yan et al., 2018). Guo et al. (2021) found that the trajectory of the moving target was perceived to be biased toward a synchronously presented landmark and away from a flashed landmark. The current study found that the size of the destination point had no significant influence on spatial orientation, whereas the landmark attraction effect was reduced in the presence of a relatively large moving target. A reasonable explanation for these phenomena is cognitive penetrability. Cognitive penetrability occurs when higher-level cognitive phenomena such as desires, experiences, and concepts directly influence perception (Newen and Vetter, 2017; Cermeño-Aínsa, 2020). We constantly perceive and process a vast richness of information, and an effective way to improve this processing is to predict the information we will receive based on previous experience (Cermeño-Aínsa, 2020). This theory has been confirmed in research on bidirectional visual pathways, and almost all areas of the visual pathway have been shown to be subject to top-down effects (Gilbert and Li, 2013). According to Newton’s law of gravitation, each particle of matter in the universe attracts every other particle with a force that is directly proportional to the product of the masses of the two particles. In our experience, compared with a small particle of matter, a relatively larger particle with the same density has a stronger gravitational attraction to other particles. This preconceived cognitive penetration led to the smaller moving targets being more easily attracted by the destination points, resulting in a larger rotation angle. In addition, attention, as a metacognitive process, is involved in cognitive penetration (Marchi, 2017). In this study, individuals dynamically perceived the movement direction of the moving target and the direction it pointed to the destination point; thus, the participants may have concentrated more attention on the moving target. This could be a possible explanation of why the size of the moving target affected spatial orientation while the size of the destination point had no significant effect.

Cognitive load, defined as the demand on cognitive control required in work tasks, is directly related to task performance (Biondi et al., 2019), and having an appropriate cognitive load is critical to efficiency and safety in work (Biondi et al., 2023). As many studies have shown, an increase in cognitive load is accompanied by an enlarged pupil size (Zekveld et al., 2014; Asadi et al., 2022; El Haj et al., 2022; Biondi et al., 2023). In the current study, the average pupil size of the participants was significantly larger when a relatively large moving target was presented. This result indicates that the spatial orientation of a moving target may impose higher cognitive load requirements on individuals when the target is relatively larger. Different opinions have been proposed to explain the mutual interaction between the cognitive processing of object characteristics and motion characteristics. Researches have found that a variety of object characteristics can also be processed in the dorsal pathway (Bettencourt and Xu, 2016; Bracci and Op de Beeck, 2016; Freud et al., 2016). The ventral and dorsal pathways potentially interact with each other, neural inputs of the ventral and dorsal cortical areas physiologically and anatomically merge (Nassi and Callaway, 2009). In addition, some scholars proposed a network model for information processing (de Haan and Cowey, 2011), they hold that the processing of object information and motion information is not based on independent linear hierarchical pathways, but rather an interconnected network model. In this study, the interaction between the cognitive processing of object characteristics and motion characteristics was accompanied by a significant difference in cognitive load. Therefore, we infer that cognitive resources activated by object characteristics processing can be used for motion characteristics processing, leading to differences in motion characteristics processing. In addition, although the velocity of the moving target was found to have no effect on spatial orientation, it had significant effects on average pupil size, fixation count, saccade count, average fixation duration, and average saccade amplitude, reflecting the difference in cognitive processing. In this study, the main purpose of using different velocities was to avoid the participants’ prediction of and preparation for motion. The use of a larger range of velocities might amplify the difference in cognitive processing and have an influence on spatial orientation.

This study provides a reference for tests of spatial orientation ability in pilot selection and training. The ability of pilots to automatically process information on motion characteristics allows them to avoid interference between the processing of information related to object characteristics and the processing of information related to motion characteristics (Yan and You, 2015). When significant visual references for estimating position and direction such as the natural horizon line are lost, spatial orientation becomes difficult (Boril et al., 2020). Avoiding the interference of object characteristics with the processing of motion characteristics is significant for maintaining spatial orientation in the absence of visual references. Eye movement technology also has great potential for flight ability test applications because of its noninvasiveness and high sensitivity (Kim et al., 2015; Vlačić et al., 2020; Jiang et al., 2021). Matton et al. suggested that task-evoked pupil size is a promising index for predicting flight training difficulty (Matton et al., 2022). High-level student pilots showed larger changes in pupil size from low-load to high-load stages, and at the low-load stage, their average pupil size was smaller (Matton et al., 2022). In contrast to traditional behavior indices, which reflect only test results, eye movement indices reflect the test process, and their application can therefore improve the efficiency of selection and training.

There are some limitations to be noted in the current study. First, the sampling was limited to male college students, and comparable research in which pilots are used as subjects may provide more meaningful results and should be the direction of future research. Second, eye movement technology cannot directly reveal brain activity during information processing, and the application of other technologies, such as magnetic resonance imaging (MRI), may be a future research direction. Third, further research is needed on the plasticity of the ability tested in this study to determine whether it is suitable for selection or training. Fourth, the sensitivity of the knob was not examined in this study, which may have a certain impact on the experimental results. Finally, our study was conducted in a laboratory environment; whether eye movement indices can be used for occupational selection and training must still be tested in practice.

## Conclusion

The present study compared the accuracy of spatial orientation and eye movement indices under conditions involving moving targets and destination points of different sizes. The results revealed that a relatively large moving target can resist the landmark attraction effect in spatial orientation and suggest that the influence of object size on motion perception and manipulation may originate from differences in cognitive resource consumption. The present study is of great significance in enriching the interaction theory of the processing of object characteristics and motion characteristics, and it provides new ideas for the application of eye movement technology in occupational selection for spatial orientation ability.

## Data availability statement

The raw data supporting the conclusions of this article will be made available by the authors, without undue reservation.

## Ethics statement

The studies involving humans were approved by the Ethics Committee of the Xijing Hospital of the Air Force Medical University (no. KY20224106-1). The studies were conducted in accordance with the local legislation and institutional requirements. The participants provided their written informed consent to participate in this study.

## Author contributions

TY and YH were responsible for the writing of this paper. LW, YL, and HW were responsible for the experimental collection of original data. TY, LW, YG, and XW were responsible for the data collection and analysis. XL, YG, and SW were responsible for the experimental design and overall planning of the research. All authors contributed to the article and approved the submitted version.
